# Functional properties of the novel PA-X endonuclease of human influenza virus

**DOI:** 10.1186/1471-2334-14-S2-P101

**Published:** 2014-05-23

**Authors:** Laura Bavagnoli, Giovanni Maga

**Affiliations:** 1 Institute of Molecular Genetics CNR, National Research Council, Pavia, Italy

## 

The PA subunit of the RNA dependent RNA polymerase of influenza virus is an endonuclease required for the cap-snatching mechanism leading to the synthesis of viral mRNA. Recently, the PA-X protein has been discovered in human influenza, which is translated from an alternative open reading frame within the PA gene coding sequence, resulting from a ribosomal frameshifting. The PA-X retains the N-terminal endonucleolytic domain of PA, but has a different and shorter C-terminal part. Two isoforms of PA-X are present in circulating human infuenza A strains. It has been proposed that PA-X degrades cellular mRNA, contributing to the attenuation of viral pathogenesis in the host. Here we express the diferent recombinant PA-X proteins from human seasonal influenza virus and show that their endonucleolytic activities have distinct substrate specificities, which depend on the structure of the RNA to be cleaved. Collectively, our results support distinct roles of PA and PA-X in the viral life cycle.

